# Concomitant therapy with Cineole (Eucalyptole) reduces exacerbations in COPD: A placebo-controlled double-blind trial

**DOI:** 10.1186/1465-9921-10-69

**Published:** 2009-07-22

**Authors:** Heinrich Worth, Christian Schacher, Uwe Dethlefsen

**Affiliations:** 1 Hospital Fürth, University Erlangen-Nürnberg, Jakob-Henle-Str. 1, D-90766 Fürth, Germany; 2 MKL Institute of Clinical Research, Pauwelsstr. 19, D-52074 Aachen, Germany

## Abstract

**Background:**

The clinical effects of mucolytics in patients with chronic obstructive pulmonary disease (COPD) are discussed controversially. Cineole is the main constituent of eucalyptus oil and mainly used in inflammatory airway diseases as a mucolytic agent. We hypothesised that its known mucolytic, bronchodilating and anti-inflammatory effects as concomitant therapy would reduce the exacerbation rate and show benefits on pulmonary function tests as well as quality of life in patients with COPD.

**Methods:**

In this double-blind, placebo-controlled multi-center-study we randomly assigned 242 patients with stable COPD to receive 200 mg of cineole or placebo 3 times daily as concomitant therapy for 6 months during winter-time. The frequency, duration and severity of exacerbations were combined as primary outcome measures for testing as multiple criteria. Secondary outcome measures included changes of lung function, respiratory symptoms and quality of life as well as the single parameters of the exacerbations.

**Results:**

Baseline demographics, lung function and standard medication of both groups were comparable. During the treatment period of 6 months the multiple criteria frequency, severity and duration of exacerbations were significantly lower in the group treated with cineole in comparison to placebo. Secondary outcome measures validated these findings. Improvement of lung function, dyspnea and quality of life as multiple criteria were statistically significant relative to placebo. Adverse events were comparable in both groups.

**Conclusion:**

Concomitant therapy with cineole reduces exacerbations as well as dyspnea and improves lung function and health status. This study further suggests cineole as an active controller of airway inflammation in COPD by intervening in the pathophysiology of airway inflammation of the mucus membrane.

**Trial registration:**

ISRCTN07600011

## Introduction

Chronic obstructive pulmonary disease (COPD) is considered to be a multi-component disease comprising structural and functional changes inside and outside the lungs. Effective medications for COPD are available and can reduce or prevent symptoms, increase exercise capacity, reduce the number and severity of exacerbations and improve health status. In common clinical use are bronchodilators as β-agonists, anticholinergic drugs and methylxanthines as well as glucocorticosteroids. The clinical effectiveness of these drugs has been shown in many controlled clinical studies [1-7].

Airway inflammation and mucociliary dysfunction in COPD patients have direct clinical consequences on the decline of lung function. As a consequence of cigarette smoking the ciliated epithelium is damaged and the mucus membrane becomes inflamed, resulting in decreased mucociliary transport leading to an accumulation of mucus within the airway so that the likelihood of recurrent respiratory infection is increased. Cineole has positive effects on the beat frequency of the cilias in the mucus membrane and has bronchodilating and anti-inflammatory effects. Therefore, it is appropriate to postulate that cineole will show positive influence on the exacerbations as well as on the lung function in COPD patients – even as concomitant therapy [8-13].

We conducted a randomised, placebo-controlled multi-center trial with the concomitant prescription of cineole – the main constituent of eucalyptus oil – in patients with stable COPD. The primary hypothesis was that cineole would decrease the number, severity and duration of exacerbations. Secondary outcome measures were lung function, severity of dyspnea and quality of life as well as relevant adverse effects.

## Materials and methods

### Enrolment of participants

Participants were recruited in the offices of 4 general practitioners and 7 specialists in pneumology in Germany. The study was carried out during the winter seasons 2003/2004 and 2004/2005 over a treatment period of 6 months in the winter and starting the enrolment in September at the earliest. The participants were 40 to 80 years of age and had airflow limitation with FEV_1 _of less than 70% and more than 30% of the predicted value (moderate to severe COPD; according to GOLD classification stages 2 and 3 of COPD) [14]. Patients with an increase of more than 15% and more than 200 ml in FEV_1 _after inhalation of β-agonists (at least 200 μg Salbutamol or equivalent) were excluded according to the definition of COPD of the German Airway-League. [15]. All patients were current smokers or ex-smokers with at least 10 pack years. Patients were excluded if they had severe medical conditions such as bronchial carcinoma, myocardial infarction, alcoholism, heart failure. All randomised patients provided written informed consent and the protocol was approved by the local Ethics Committees at each of the 11 participating centres.

### Treatment groups

242 patients were randomly assigned to one of the two treatment groups with stratification according to the clinical centres. All patients were given the necessary dose of capsules each containing 100 mg cineole or no active ingredient. For each group 2 capsules 3 times daily were prescribed resulting in a dose of 600 mg per day for the cineole or no cineole for the placebo group as concomitant therapy. Patients were instructed to take the capsules half an hour before meal so that they could not recognize the smell of cineole. Capsules with active substance and placebo looked absolutely identical and were sealed in blister stripes.

The diagnosis of COPD was confirmed according to the current guidelines of "Global Initiative for Chronic Obstructive Lung Disease" (GOLD). Frequency, duration, severity and symptoms of exacerbations were defined according to the literature [16-18]. An exacerbation was documented when the duration was more than 3 days or a complex of at least 2 respiratory adverse events with a duration of more than 3 days occurred. Exacerbation severity was defined as: mild (Score = 1, increased need for basic medication of COPD which the individual can manage in its own normal environment), moderate (score = 2, increased need for medication and he/she feels the need to seek additional medical assistance) and severe (score = 3, patient recognise obvious and/or rapid deterioration in conditions requiring hospitalisation). Details for the number, duration and severity as well as treatment and symptoms of exacerbations were recorded in the patient's diary for each day. Since the most relevant differentiation for exacerbations are frequency, duration and severity, the multiple criteria were combined as primary outcome measures for the statistical evaluation.

Secondary outcome measures were the single parameters of the exacerbation as well as lung function, symptoms and quality of life. Spirometric measurements were carried out before the beginning of the study determining reversibility of the airflow limitation by inhalation of short acting β2-agonists to assure that the reversibility of lung function was less than 200 ml or 15%. Spirometric measurement included determination of forced expiratory volume in 1 second (FEV_1_), forced vital capacity (FVC) and vital capacity (VC) at the beginning and after 3 and 6 months. Additionally, symptoms score were determined for dyspnea (scores: 0 = caused no problems, 1 = caused occasionally problems, 2 = caused a lot of problems, 3 = the most important problem the patient had), weekly frequency of dyspnea (scores: 0 = no day was good, 1 = 1–2 days were good, 2 = 3–4 days were good, 3 = nearly every day was good, 4 = every day was good), general conditions (scores: 0 = good, 1 = impaired, 2 = bad, 3 = very bad, 4 = unbearable), cough (scores: 0 = never, 1 = rarely, 2 = occasionally, 3 = often, 4 = very often, 5 = nearly continuously).

Diagnosis-related quality of life was determined according to the "St. George's Respiratory Questionnaire" [19].

### Visits and Randomization

Before randomisation we ascertained the patients' eligibility and conducted spirometry. After the randomisation the following parameters were recorded: height, weight, age, time since first symptoms for the diagnosis of COPD, documentation of allergies, concomitant disease, smoking habits (documentation of pack years), number of exacerbations in the year before during winter time, determination of quality of life and current maintenance therapy. The following control visits were carried out after 1, 2, 3, 4, 5 and 6 months recording exacerbations since last visit, frequency of dyspnea, characterisation of dyspnea, hypersecretion and cough as well as adverse events, compliance and change of therapy. Spirometry was carried out at the beginning of the study as well as after 3 and 6 months of treatment. Quality of life was determined at the beginning and at the end of the study.

### Statistical analysis

The proposed sample size for the present trial was 240 patients for both treatment groups. The sample size was chosen to detect a minimum difference of 15% of exacerbations after 6 months of treatment. Analysis of efficacy was performed with the intention-to-treat-population including all eligible patients who received at least one dose of medication and had at least one follow-up visit. The number of all exacerbations, duration of exacerbations, degree of severity of exacerbation recorded in patients diary during the 6 months treatment period were summarised and the sums compared according to Wei-Lachin's directional test of multiple criteria (equally weighted) [20]. Additionally, single parameters characterising the exacerbations and dyspnea were analyzed exploratory as secondary outcome measures at multiple endpoints according to Wei-Lachin validating the sum-formation. The Wilcoxon-Mann-Whitney-U Test was used for all other secondary outcome measures. Data are expressed as mean values (with SD) and all tests were two-tailed. P-values of 0.05 or less were considered to indicate statistical significance.

## Results

A total of 242 patients were randomised and received at least one dose of study medication. 22 patients were excluded from the statistical analysis of efficacy because they did not meet the requirements of the GOLD guidelines since FEV_1_/VC was > 0.7. 220 patients were eligible according to the GOLD guidelines having COPD of stage II and III. The two treatment groups were well matched with respect to baseline characteristics (table 1). The mean age of the participants at entry was 62 years in both groups. The mean duration of COPD of 13 years as well as 31 pack-years and the basic medication (i.e. ICS, β-agonists, anticholinergics and theophylline) were balanced between the two groups (table 2). Medication was not changed during the treatment period except in occurrence of exacerbations. The baseline lung function and reversibility in both groups were comparable. Treatment compliance was determined by counting the study medication at each visit and was found high and comparable across the treatment groups.

**Table 1:** Base Line Characteristics of the Patients*

***CHARACTERISTIC***	**PLACEBO** **(N = 110)**	**CINEOLE** **(N = 110)**
Age – yr		
Mean	62.5 ± 10.2	62.2 ± 9.1
Range	40 – 79	41 – 79
		
Sex – M/F	75/35	66/44
		
Weight – kg		
Mean	79 ± 16	79 ± 14
Range	48 – 120	52 – 113
		
Height – m		
Mean	1.71 ± 8.8	1.70 ± 8.0
Range	1.48 – 1.90	1.54 – 1.92
		
Years since appearance of COPD		
Mean	12.8 ± 9.7	13.6 ± 10.9
Range	1 – 56	1 – 59
		
Severity of COPD [number of patients]		
Moderate COPD (II)	69	68
Severe COPD (III)	41	42
		
FEV_1_/VC [%]	58.0	58.7
		
FEV_1 _[l]	1.61 ± 0.5	1.62 ± 0.5
		
FEV_1 _% predicted value	54.4	54.9
		
Reversibility (increase of FEV_1 _after inhalation of β-agonist) [%]	5.5	4.5
		
Allergies (number)	10	9
		
Pack years	31.5 ± 19	31.3 ± 16

* Plus – minus values are means ± SD.

**Table 2:** Constant concomitant therapy

***THERAPY***	**PLACEBO** **(N = 110)**	**CINEOLE** **(N = 110)**
Inhaled β-agonists* (LABA and SABA)	83	88
Inhaled anticholinergics	29	35
Inhaled corticosteroids (ICS)	27	25
Theophylline	38	32

* including combinations

### Primary outcome measures

#### Exacerbations

At baseline the mean exacerbation rate was 3.2 in both groups during the previous year. The number of patients with exacerbations during the treatment period in the cineole group was 31 patients (28.2%) and 50 patients (45.5%) in the placebo group. As primary outcome measure the sum of exacerbations for frequency, duration and severity at all 6 following visits as composite endpoint (equally weighted) were calculated according to the Wei-Lachin Test procedure for multiple criteria and showed a statistically significant difference for the primary outcome measure between both treatment groups (p = 0.0120) Table 3. Calculating these single parameters alone exploratory according to Mann-Whitney-U it could be proven that they were statistically significant too (i.e. for frequency 0.0069 an, duration 0.0210 and for severity 0.0240). Validating these results by Wei-Lachin-Test procedure for multiple endpoints for the number, the degree and the severity of exacerbations, the degree during the 6-month treatment the differences between both treatment groups were statistically significant (p = 0.0016, 0.0031 and 0.0025) which underlines higher sensitivity of this test-procedure. Medication of the exacerbations with additionally applied corticosteroids occurred in 17 cases in the cineole and in 25 cases in the placebo group which was not statistically significant different.

**Table 3:** Mean of sum of number, duration and severity of exacerbations during 6 months of treatment with cineole or placebo*

	**PLACEBO**	**CINEOLE**	
	SCORE	SCORE	P VALUE†
**Sum of exacerbations (number)#**	0.9 ± 1.46	0.4 ± 0.82	0.0069
**Sum of duration (days)#**	5.7 ± 8.9	4.0 ± 10.9	0.0210
**Sum of severity (score)#**	1.4 ± 2.2	0.8 ± 1.5	0.0242
**Summarized parameter** **(directional test)**			0.0120

* Plus – minus values are means ± SD.

† P-Values are for the comparison between the two groups.

# The sum of the exacerbation parameters is calculated by addition of the documented parameters at all visits.

### Secondary outcome measures

#### Lung function

Patients only discontinued inhaled β_2_-agonist prior to spirometry testing. After inhalation of a β_2_-agonist the reversibility of lung obstruction (increase of FEV_1_) at the start of the study was 4.5% in the cineole group and 5.5% in the placebo group (table 1). After 6 months of treatment the mean FEV_1 _increased by 78 ml (4.7%) in the cineole group (table 4). The mean differences between both groups were not statistically significant (p = 0.0627). After 6 months of treatment an increase of FVC by 62 ml (2.7%) after cineole therapy and a decline of 25 ml (1.1%) after treatment with placebo was determined. The difference concerning change of FVC and VC between both treatment groups was not clinically relevant.

**Table 4:** Secondary outcome measures during 6 months Of treatment with cineole or placebo for change of lung function and dyspnea symptoms *

**LUNG FUNCTION AND SYMPTOMS**	**PLACEBO**			**CINEOLE**			
	BASE LINE	3 MONTHS	6 MONTHS	BASE LINE	3 MONTHS	6 MONTHS	**p-Value**
**FEV 1 [l]**	1.61 ± 0.5	1.62 ± 0.5	1.61 ± 0.5	1.62 ± 0.5	1.67 ± 0.5	1.70 ± 0.6	0.0627**†**
**FVC [l]**	2.23 ± 0.8	2.25 ± 0.8	2.22 ± 0.7	2.33 ± 0.8	2.36 ± 0.9	2.36 ± 0.9	0.2409**†**
**VC [l]**	2.81 ± 0.8	2.71 ± 0.7	2.68 ± 0.8	2.80 ± 0.8	2.73 ± 0.8	2.72 ± 0.9	0.2060**†**
**Trouble in breathing #**	1.8 ± 0.9	2.1 ± 0.9	2.2 ± 1.0	1.9 ± 0.9	2.4 ± 1.0	2.5 ± 1.1	0.0103**&**
**Dyspnea in the morning $**	1.1 ± 0.7	0.9 ± 0.7	0.7 ± 0.7	1.1 ± 0.8	0.7 ± 0.7	0.5 ± 0.6	0.0466**&**
**Dyspnea at rest $**	0.7 ± 0.7	0.4 ± 0.6	0.4 ± 0.6	0.6 ± 0.6	0.3 ± 0.5	0.3 ± 0.5	0.0156**&**
**Dyspnea during exercise $**	2.0 ± 0.6	1.8 ± 0.7	1.7 ± 0.8	2.0 ± 0.6	1.7 ± 0.7	1.5 ± 0.9	0.1252**&**

* Plus – minus values are means ± SD. Higher scores on the symptoms-sum-score indicate more disease activity.

**† **P-Values are for the comparison of the changes from base line to 6 months between the two groups.

**&**P-Values for the comparison are calculated by the multiple criteria calculation of 6 visits by Wei-Lachin between the two groups.

**# **Scores: 0 = no day was good, 1 = 1–3 days were good, 2 = nearly every day was good, 3 = every day was good in a week.

**$ **Scores: 0 = did not cause any problems, 1 = sometimes caused problems, 2 = caused a lot of problems, 3 = the most important problem the patient had

#### Dyspnea

The differences between both groups after 6 months of treatment are summarised in table 4. The baseline dyspnea scores in the morning, trouble in breathing, dyspnea at rest and dyspnea during exercise were similar in both groups, indicating a moderate level of a dyspnea for most patients at the beginning of the treatment period. Calculating the values at all 6 visits at multiple endpoints the difference between both treatment groups were statistically significant for trouble in breathing, dyspnea in the morning and dyspnea at rest. Dyspnea during exercise did not show a statistically significant difference between treatment groups.

#### Quality of life

At 6 months the mean improvement of SGRQ total symptom score was -9.1 after treatment with cineole and -4.1 after treatment with placebo (table 5). The difference between treatment groups was not statistically significant (p = 0.0630). The improvement of the symptom score was statistically significant (p = 0.0224). The differences of changes of activity score and impact score were not statistically significant between the two groups.

**Table 5:** Secondary outcome measures for saint george's respiratory questionnaire (sgrq): Change of total symptom score, symptom score, activity score and impact score During 6 months of treatment with cineole or placebo*

**SGRQ SCORES**	**PLACEBO**		**CINEOLE**		**P VALUE †**
	BASE LINE	6 MONTHS	BASE LINE	6 MONTHS	
**Symptom score**	57.4 ± 20.2	48.5 ± 24.9	57.3 ± 20.4	43.8 ± 24.3	0.0224
**Activity score**	53.4 ± 21.9	50.0 ± 24.8	52.1 ± 20.5	43.5 ± 22.4	0.2032
**Impact score**	37.2 ± 20.9	33.9 ± 23.3	35.8 ± 20.2	27.4 ± 19.2	0.1126
**TOTAL SYMPTOM SCORE**	45.6 ± 18.9	41.3 ± 22.5	44.4 ± 17.8	34.5 ± 18.9	0.0630

* Plus – minus values are means ± SD. Lower scores on the scores indicate higher quality of life.

† P-Values are for the comparison of the changes in scores from base line to 6 months treatment between the two groups.

### Multiple criteria

Symptomatic of COPD patients is characterized by lung function, dyspnea and quality of life. In order to include these relevant secondary outcome measures together we used these parameters equally weighted as multiple criteria. The increase of FEV1, amelioration of dyspnea and improvement of total score of "SGRQ" were calculated according to the Wei-Lachin Test procedure for multiple criteria and showed a statistically significant difference for the relevant secondary outcome measure between both treatment groups (p = 0.0024).

#### Other findings

Concomitant therapy with β-agonists and anticholinergics, corticosteroids or combinations and methylxanthines in both groups were comparable (table 2). The global assessment of efficacy showed a significant difference, which correlated with the amelioration of clinical findings after treatment with cineole.

### Side effects

All patients receiving the study medication (including those with FEV_1_/VC > 0.7) were included in the safety examination. During treatment side effects were seen in 22 patients whereas in 17 cases adverse events were not related to the study medication. In the placebo group 11 adverse events were estimated not being related to the study medication whereas 2 cases were interpreted as being related to the study medication (heartburn). During treatment with cineole 9 cases of adverse events were reported whereas 6 adverse events were reported not being related to the study medication. In 3 patients (nausea, diarrhoea, heartburn) the adverse events were estimated being related to the study medication. The difference between the two treatment groups was neither clinically relevant nor statistically significant. Safety examinations of the global assessment showed no difference between the two treatment groups. During the 6 months of treatment compliance was good in all patients.

## Discussion

Patients with COPD experience exertional breathlessness caused by bronchoconstriction, mucous secretion, edema of the airway wall and loss of attachments to the terminal airways [14]. Hence pharmacological therapy has focused on the treatment of airway obstruction and inflammation to improve symptoms primarily dyspnea as well as health status. Bronchodilators are the mainstay of pharmacotherapy for patients with COPD. On the other hand it is well known that mucociliary dysfunction has direct clinical implications. Mucus is beneficial in normal quantities but in case of mucus hypersecretion when cilia fail, the mucus pool allows bacterial colonisation. The presence of pooled bacteria results in the release of bacterially-derived toxins that destroy the underlying epithelium and trigger a neutrophilic response [21]. Taking into account the known pharmacological effects of the defined natural product cineole it was assumed that this compound might be beneficial for patients with COPD.

Exacerbations have been shown to be reduced in various studies evaluating treatment with inhaled β-agonists or corticosteroids or combinations. The major finding of the present study is that cineole provides a significantly greater reduction of frequency, duration and severity of exacerbations than placebo. The exacerbations were analyzed as multiple criteria for the relevant specifications frequency, duration and severity. The result for the primary outcome measure could be validated exploratory for the single parameters showing a statistical significant difference too. Therefore, both testing procedures are valid whereas the testing multiple criteria is more sensitive. This proof of efficacy is an important contribution to the known pharmacological properties of cineole which therefore is not a mucolytic agent only. The result of this study suggests important new evidence of superior therapeutic efficacy of additional therapy with cineole to better control COPD exacerbations compared to the currently recommended combined therapy with ICS and LABA. Furthermore, additional therapy with cineole may positively interact with anti-inflammatory activity of recommended airway therapies in COPD and may serve to protect airways from other environmental agents.

In general, quality of life deteriorates slowly in patients with COPD. During the period of 6 months treatment of this study we observed a decrease of the scores of SGRQ in both treatment groups. The reason for this finding in the placebo group, too, seems to be due to patients receiving better medical attention when involved in clinical trials. The higher rate of exacerbations in the winter before the study began in both treatment groups is due to the same reason. Our present data with cineole therapy underline a greater improvement than after treatment with placebo. Differences in change of FEV_1 _were not statistically significant (p = 0.0627). These findings correlate with a decline of FEV_1 _in the placebo group of 0.4% and an increase of 4.8% in the cineole group. This value is nearly identical with the increase of FEV_1 _after the inhalation of β-agonists, when testing the reversibility, before concomitant therapy with cineole started for six months.

## Conclusion

These collective findings underline that cineole not only reduces exacerbation rate but also provides clinical benefits as manifested by improved airflow obstruction, reduced severity of dyspnea and improvement of health status. Therefore, cineole can provide a useful treatment option for symptomatic patients with COPD in addition to treatment according to the guidelines. These results have to be seen in context with socio-economic aspects. As COPD is an extremely costly disease and a cause of major financial and social burden concomitant therapy with cineole can be recommended, especially due to the lack of relevant side effects and relatively low cost. The results of our study provide good evidence that cineole will show benefits as additional therapeutic regimen in patients with COPD. These findings correspond to the interpretation of the efficacy-study with Carbocysteine but not with Acetylcysteine, because this medication did not show a significant reduction of exacerbations [22,23].

## Competing interests

The authors declare that they have no competing interests.

## Authors' contributions

The study was designed and the protocol developed by HW, C S and UD. CS worked as principal investigator. Statistical analysis was carried out by UD. The results were interpreted by HW, CS and UD. All authors gave substantial critical input in revising the manuscript.
